# Differential Effects of Biomimetic Thymine Dimers and Corresponding Photo-Adducts in Primary Human Keratinocytes and Fibroblasts

**DOI:** 10.3390/biom14121484

**Published:** 2024-11-21

**Authors:** Rosanna Monetta, Denise Campagna, Valeria Bartolocci, Alessio Capone, Massimo Teson, Silvia Filippi, Sofia Gabellone, Davide Piccinino, Raffaele Saladino, Elena Dellambra

**Affiliations:** 1 Laboratory of Molecular and Cell Biology, Istituto Dermopatico Dell’Immacolata (IDI-IRCCS), 00167 Rome, Italy; ros.monetta88@gmail.com (R.M.); denise.campagna20@gmail.com (D.C.); v.bartolocci@idi.it (V.B.); a.capone@idi.it (A.C.); m.teson@idi.it (M.T.); 2 Laboratorio di Genetica dell’Invecchiamento, Dipartimento di Scienze Ecologiche e Biologiche, Università degli Studi della Tuscia, Largo dell’Università snc, 01100 Viterbo, Italy; silvia.filippi@unitus.it; 3 IRCCS Istituto Romagnolo per lo Studio dei Tumori “Dino Amadori”—IRST Srl, 47014 Meldola, Italy; sofia.gabellone@irst.emr.it; 4 Centro Integrato di Ateneo, Sezione Centro Grandi Attrezzature, Università degli Studi della Tuscia, Largo dell’Università snc, 01100 Viterbo, Italy; d.piccinino@unitus.it; 5 Dipartimento di Scienze Ecologiche e Biologiche, Università degli Studi della Tuscia, Via S Camillo de Lellis, 01100 Viterbo, Italy; saladino@unitus.it

**Keywords:** thymine dimers, keratinocytes, fibroblasts, skin, UVB radiation

## Abstract

UVB radiation induces DNA damage generating several thymine photo-adducts (TDPs), which can lead to mutations and cellular transformation. The DNA repair pathways preserve genomic stability by recognizing and removing photodamage. These DNA repair side products may affect cellular processes. We previously synthesized novel thymine biomimetic thymine dimers (BTDs) bearing different alkane spacers between nucleobases. Thus, the present study investigates whether novel BTDs and their TDPs can modulate DNA damage safeguard pathways of primary keratinocytes and fibroblasts using 2D and 3D models. We found that the p53/p21^waf1^ pathway is activated by BTDs and TDPs in primary cells similar to UVB exposure. Compound **1b** can also induce the p53/p21^waf1^ pathway in a 3D skin model. However, BTDs and TDPs exhibit distinct effects on cell survival. They have a protective action in keratinocytes, which maintain their clonogenic ability following treatments. Conversely, compounds induce pro-apoptotic pathways in fibroblasts that exhibit reduced clonogenicity. Moreover, compounds induce inflammatory cytokines mainly in keratinocytes rather than fibroblasts. Matrix metalloproteinase 1 is up-regulated in both cell types after treatments. Therefore, BTDs and TDPs can act in the short term as safeguard mechanisms helping DNA damage response. Furthermore, they have distinct biological effects depending on photodamage form and cell type.

## 1. Introduction

Prolonged exposure to ultraviolet (UV) rasdiation may cause DNA damage, which subsequently leads to mutations, cell death, or cellular transformation. In particular, UV photons excite thymine pyrimidine bases, which produces thymine photo-adducts (TDPs), including Cyclobutane Pyrimidine Dimer (CPD), Dewar valence isomer (DVI), and Pyrimidine-(6-4)-Pyrimidone (6-4 PP) [1]. The primary TDPs generated after UVB exposure are CPDs produced from a [2+2] cycloaddition reaction, typically involving thymines neighboring each other on the same DNA strand. CPDs influence the DNA processing mechanisms and contribute to mutation of absorbing properties [1,2]. DVIs are products of photoisomerization of 6-4 PP under UVA and UVB irradiation and are generated in lower yield than CPDs [2].

Cutaneous absorption of UVB occurs primarily in keratinocytes, causing DNA and protein damage. To prevent DNA mutations, cells engage sophisticated DNA repair mechanisms, such as nucleotide excision repair (NER), encompassing photodamage recognition and excision [3,4]. If DNA damage is mild, the cell undergoes cell cycle arrest by p53/p21^waf1^ pathway activation to allow repair. This process involves the excision and release of photo-damaged DNA, which encompasses approximately a 30 nucleotide(nt)-long modified oligomer. The excised DNA region is then resynthesized and the cell re-enters the cell cycle. However, persistent UV-induced DNA lesions can trigger specific p53-mediated cellular responses leading to apoptosis or senescence, which are two tumor suppressor mechanisms preventing damaged cells from undergoing neoplastic transformation [5,6].

Recent studies have indicated that DNA repair side products (e.g., damaged oligomers and bases) may form complexes with related repair enzymes [3,7]. For instance, excised oligonucleotides containing thymine dimers are released from DNA in a complex with the repair factors TFIIH, XPG, and to a small extent XPF. Subsequently, they are slowly dissociated from TFIIH in an ATP-dependent manner, can be bound by Replication Protein A (RPA), and be subject to a limited nucleolytic breakdown. Once released from RPA, oligonucleotides undergo further degradation by cellular nucleases [3,8,9,10]. The lesion-containing oligomers can have a biological function by activating specific intracellular signaling pathways. They can bind to DNA at a nonspecific binding site of the transcription factor p53, stimulating its specific binding to target genes. Thus, it has been proposed that UVB radiation may activate a p53-mediated DNA damage response through the association between removed photo-products and p53 itself [3], suggesting that oligomers may act as signals for coordinating cellular response to DNA damage. Simple thymine dimers (TDs) bearing an alkyl spacer between the two bases have been shown to mimic the biological effects of UV irradiation in cultured skin cells, at least in part, through activation of p53 and p21^waf1^ [11]. Without an initial DNA injury, TDs activate the p53 pathway and make cells more capable of responding to successive DNA damage. In particular, they can stimulate the rapid repair of damaged DNA and improve both cell survival and clonogenicity [12]. Topical application of TDs resulted in DNA photoprotection, reducing the development of skin cancer in UV-irradiated mice [13,14,15].

Recently, we reported the synthesis of a panel of biomimetic thymine dimers (BTDs) that differ in the length of the alkyl spacer between the two nucleobases and can inhibit the growth of melanoma cell lines [16]. The present study aims to investigate whether BTDs, including CPD and DVI photo-adducts, can have UV-mimetic effects on primary keratinocytes and fibroblasts using 2D and 3D models. The focus is on evaluating the impact of BTDs on DNA damage safeguard pathways. Since sun exposure can lead to altered inflammatory and immune responses, which induce photosensitivity, the contribution of BTDs to cell inflammation is also assessed.

## 2. Materials and Methods

### 2.1. Synthesis of Thymine Biomimetic Dimers

The BTDs **1a**–**b** were synthesized by applying the protocol as described in [16]. Briefly, thymine was dissolved in dry CH3CN and N,O-bis-(trimethylsilyl)-acetamide (BSA) at 25 °C and after 4.0 h. Then, the mixture was evaporated to yield O,O-bis-trimethylsilyl thymine in quantitative yield. This intermediate was dissolved in the appropriate alkyl-dibromide and dry piperidine at 80 °C under an argon atmosphere for 4 days. The thymine dimers were purified by flash chromatography.

### 2.2. Preparation of Photo-Adducts CPD and DVI

CPD (**2a**–**b**) and DVI (**3a**–**b**) were prepared from compounds **1a**–**b** by applying the protocol described in [16]. Briefly, compounds **1a**–**b** (0.20 mmol) were dissolved in MilliQ water–acetone mixtures (40% *v*/*v*, 100 mL) in a Pyrex flask equipped with a condenser, degassed by bubbling with high-purity argon and irradiated with a Haerus source for 2 h under argon atmosphere at 25 °C. The crude compound was purified by flash chromatography to yield CPD (**2a**–**b**) and DVI (**3a**–**b**). Aliquots of the appropriate compound (1.0 mmol) were solubilized in dimethyl sulfoxide (DMSO).

### 2.3. Cell Cultures of Primary Keratinocytes and Fibroblasts

Human keratinocytes and fibroblasts were obtained from skin biopsies of healthy donors. Procedures were approved by the Ethical Committee of IDI-IRCCS, Rome, Italy.

Primary keratinocytes were grown on a feeder layer of lethally irradiated 3T3-J2 cells as previously described [17,18]. Primary fibroblasts were cultured as described previously [17]. Primary keratinocyte and fibroblast subconfluent cultures were used for further experiments. Cells were treated with BTDs and TDPs at 100 μM for 4, 6, and 24 h in both keratinocytes and fibroblasts. UVB irradiation (100 mJ/cm^2^) was used as the positive control.

### 2.4. The 3D Model

Skin equivalents were prepared as described previously [17]. Briefly, to obtain a collagen gel, calf skin type I collagen (Symatese Biomateriaux) was mixed with 1 × 106 primary fibroblasts. After 3 days, 5 × 10^4^ keratinocytes were plated on each collagen gel. Keratinocyte cultures were performed in submerged conditions for 7 days; then, cultures were raised at the air–liquid interface for a further 7 days. The 3D dimensional models were treated with BTDs for 24 h. The specimens were fixed in paraformaldehyde (4% in PBS) and embedded in paraffin for immunohistochemical analysis.

### 2.5. Colony-Forming Efficiency (CFE) Assay

A colony-forming efficiency (CFE) assay was performed as previously described [17,19]. Briefly, 1000 keratinocytes or fibroblasts were plated on 60 mm dishes. Colonies were fixed with 3.7% formaldehyde 14 days later, and stained with 1% Rhodamine B or Blue Coomassie, respectively. Total colonies were calculated as a percentage of total plated cells. For keratinocytes, paraclones (composed of large and flattened, terminally differentiated cells) were calculated as a percentage of total colonies.

### 2.6. Quantitative RT-PCR

RNA was extracted from cells by TRIzol (Invitrogen, Carlsbad, CA, USA). Total RNA was reverse-transcribed by an oligo(dT) primer (Aurogene, Rome, Italy). mRNA levels were analyzed by a QuantiTect SYBR Green-PCR kit (Qiagen, Hilden, Germany) with an ABI PRISM 7000 (Applied Biosystems, Foster City, CA, USA). mRNA levels were normalized by the GUSB gene as the housekeeping gene. Primers are shown in Table S1. Relative quantities of mRNA expression levels were calculated according to the 2^−ΔΔCt^ method.

### 2.7. Immunoblot Assay

Subconfluent keratinocytes were extracted as previously described [17], and equal amounts of proteins were electrophoresed on 12.5% SDS polyacrylamide gels. Immunoblotting was performed using the following antibodies: anti-p53 (DO-1) and anti-p21^waf1^ (187) from Santa Cruz Biotechnology, Inc. (Santa Cruz, CA, USA); anti-fosfo-istone H2AX (Ser139), clone JBW301, from Merk (Darmstadt, Germany). Protein expression levels were evaluated by densitometric analysis and then normalized to GAPDH levels.

### 2.8. EdU Assay

The Click-iT 5-ethynyl-2’-deoxyuridine Alexa Fluor 488 Imaging Kit (Thermo Fisher, Waltham, MA, USA) was used. Treated cultures were incubated with 5-ethynyl-2’-deoxyuridine (EdU) for 2 h. All nuclei (counterstained with Dapi) of 10 random fields (around 500 nuclei) per treatment were counted. The results are reported as EdU-positive nuclei/Dapi-stained nuclei. For fibroblasts, the percentage of more intense EdU nuclei was calculated on total EdU-positive nuclei.

### 2.9. Immunohistochemistry

Immunohistochemistry was carried out as described previously [17], using the following antibodies: anti-p53 (DO-1) and anti-p21^waf1^ (F5) from Santa Cruz Biotechnology, Inc. (Santa Cruz, CA, USA).

### 2.10. MTT Viability Assay

MTT viability assay was carried out according to Reference [16]. Briefly, after treatments with BTDs, MTT solution (0.5 mg/mL) was added for 3 h at 37 °C. Optical density detection was performed by DTX880 Multimode Detector (Beckman Coulter, Brea, CA, USA) with 630 nm and 570 nm filters. The percentage of cell viability was calculated as % cell viability = 100 − % cell cytotoxicity. 

### 2.11. Statistical Analyses

Statistical analysis was performed using GraphPad Prism 9 software. The results were expressed as mean and standard deviation (SD). Two-tailed Student’s *t*-test was carried out to determine whether differences between controls and treatment samples reached statistical significance (*p* < 0.05). UVB treatments were compared to the control (C), whereas BTD treatments were compared to cells treated with DMSO (C2).

## 3. Results

### 3.1. Effects of BTDs/TDPs on p53/p21waf1 Signaling Pathway Activation in Primary Keratinocytes and Fibroblasts

The p53 protein level and activity are up-regulated after genotoxic injury, whereas mRNA levels have been differentially reported as down-regulated or up-regulated depending on the cell type [11]. UVB increases p53 accumulation by post-translational modifications. In turn, p53 positively regulates the expression of p21^waf1^, a cyclin-dependent kinase inhibitor leading to cell-cycle arrest [6,20].

**Figure 1:**
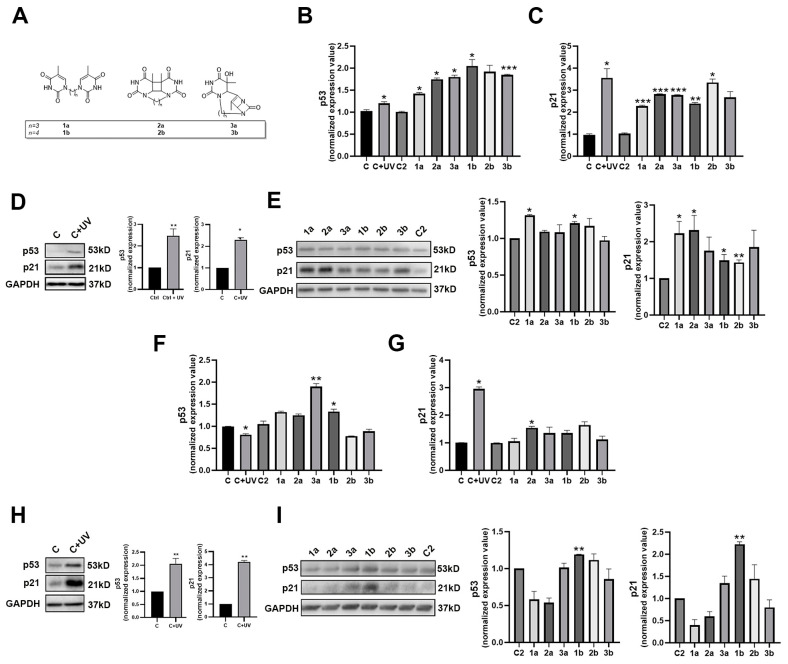
Effects of BTDs/TDPs on p53/p21waf1 signaling pathway activation in primary keratinocytes and fibroblasts. Structures of biomimetic thymine dimers (BTDs) (**1a**–**b**) and thymine photo-adducts, CPD (**2a**–**b**) and DVI (**3a**–**b**), used in the present study (**A**). Expression values of p53 and p21^waf1^ transcripts (**B**,**C**) in primary human keratinocytes after UVB and compound treatments. Immunoblots and densitometric values of p53 and p21^waf1^ protein expression in primary human keratinocytes after UVB (**D**) and compound (**E**) treatments. Expression values of p53 and p21^waf1^ transcripts (**F**,**G**) in primary human fibroblasts after UVB and compound treatments. Immunoblots and densitometric values of p53 and p21^waf1^ protein expression in primary human keratinocytes after UVB (**H**) and compound (**I**) treatments. Original western blots can be found at Supplementary Figures S2 and S3. Significance was assessed by Student’s *t*-test (* *p* < 0.05, ** *p* < 0.01, and *** *p* < 0.001).

Thus, the p53/ p21^waf1^ signaling pathway was studied by treating primary human keratinocytes and fibroblasts with BTDs (**1a**–**b**) and their photo-adducts, CPD (**2a**–**b**) and DVI (**3a**–**b**), which were dissolved in DMSO (Figure 1A). BTDs and respective TDPs were synthesized by a previously reported photochemical protocol [16]. Reference conditions included untreated primary keratinocytes and fibroblasts (C), DMSO-treated keratinocytes and fibroblasts (C2), and UVB-treated (100 mJ/cm^2^) keratinocytes and fibroblasts (C + UV). BTDs and TDPs were not toxic (MTT assay) at doses 50–100 μM in both keratinocytes (Figure S1) and fibroblasts [16] treated for 24 h.

Transcript and protein expression levels of p53 and p21^waf1^ were assessed by RT-qPCR and immunoblot, respectively. RT-qPCR analyses demonstrated a significant up-regulation of both p53 and p21^waf1^ transcripts in primary keratinocytes exposed to UVB, as well as to compounds **1a**–**b**, **2a**–**b**, and **3a**–**b** compared to controls (Figure 1B,C). Concurrently, UVB treatments significantly increased p53 and p21^waf1^ protein levels in these keratinocytes (Figure 1D). BTDs and TDPs similarly induced p21^waf1^ protein expression to a degree comparable to UVB (Figure 1E).

Primary fibroblasts exposed to UVB mainly exhibited a significant up-regulation of p53 and p21^waf1^ transcripts (Figure 1F,G). However, the effect of BTD compounds on p53 and p21^waf1^ transcription in fibroblasts was less evident compared to keratinocytes (Figure 1F,G). UVB exposure significantly induced p53 and p21^waf1^ protein accumulation in primary fibroblasts (Figure 1H). Similarly, compounds **1**–**3b** increased the levels of both proteins, with dimer **1b** inducing a notable accumulation of these proteins (Figure 1I).

Thus, the p53/p21^waf1^ pathway is activated in primary skin cells by BTDs and corresponding TDPs. Specifically, the effector p21^waf1^ is induced in keratinocytes by the complete panel of compounds, whereas it is mainly induced in fibroblasts by compounds **1**–**3b**.

### 3.2. Effects of BTDs/TDPs on DNA Repair and Survival Pathways in Primary Keratinocytes and Fibroblasts

A finely tuned and balanced expression of proteins involved in DNA repair and survival pathways can allow for a gradual response to DNA damage [21]. Three phosphatidylinositol 3’ kinase-related kinases (PIKKs) (i.e., Ataxia telangiectasia-mutated (ATM) protein, the AT and Rad3-related protein (ATR), and DNA-dependent protein kinase (DNA-PK)) are master regulators of the DNA damage response that activates the p53 response pathway [22]. Based on cell type as well as the seriousness of the DNA damage, the p53/p21^waf1^ pathway can promote transient cell-cycle arrest and repair, or apoptosis [23].

Beyond p53, PIKKs can phosphorylate the histone H2AX at Ser139. It plays a key role in the response to DNA damage for the assembly of DNA repair proteins as well as for the activation of cell-cycle checkpoints to maintain genomic integrity [22].

Proliferating Cell Nuclear Antigen (PCNA) is a p53-induced protein acting both in DNA replication and repair [24]. PCNA is a critical protein for organizing and orchestrating events at the replication fork for DNA synthesis. Moreover, PCNA has a key role in DNA repair by regulating the access of DNA repair proteins to damaged sites and favoring the de novo synthesis by DNA polymerases to fill the excision gap. Notably, p21^waf1^ has different effects on the function of PCNA in DNA replication and repair. After a genotoxic injury, p21^waf1^ can mediate growth arrest by inhibiting cyclin-dependent kinase/cyclin complexes and suppress S-phase DNA synthesis by associating with PCNA to enable active DNA repair [24,25]. 

P53 regulates cell survival by balancing the levels of Bcl-2 family members within the cell. Following several stresses, p53 promotes apoptosis by inducing the expression of the pro-apoptotic target Bcl-2-associated X protein (Bax), leading to the activation of apoptosomes and caspases. In parallel, p53 also exerts its pro-apoptotic effect by down-regulating anti-apoptotic proteins such as B-cell lymphoma 2 (Bcl-2). Apoptosis ensures the elimination of damaged or stressed cells and prevents potential oncogenesis [20,23]. However, beyond their canonical role in apoptosis regulation, the Bcl-2 family members broadly affect multiple DNA repair systems and, therefore, the maintenance of genome integrity. Bcl-2 inhibits all DNA repair systems and slows the repair of CPDs. Conversely, Bax favors NER activity [21].

**Figure 2:**
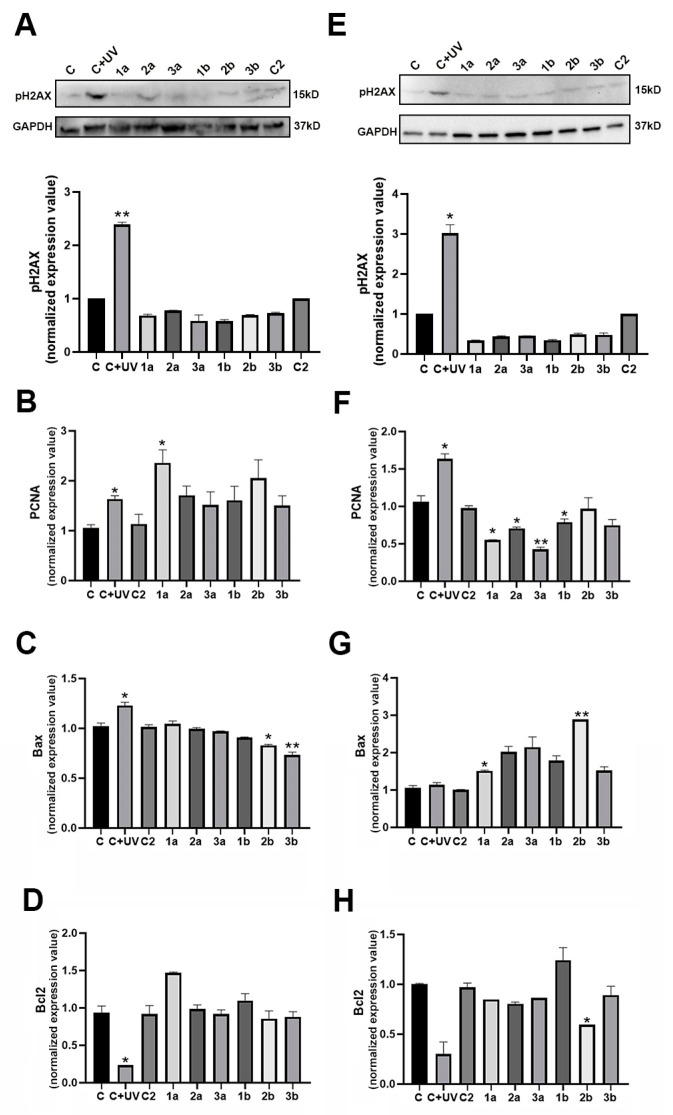
BTDs/TDPs and survival pathways in primary keratinocytes and fibroblasts. Immunoblots and densitometric values of pHA2X expression in primary human keratinocytes (**A**) and fibroblasts (**E**) after UVB and compound treatments. Expression values of PCNA, Bax, and Bcl-2 transcripts in primary human keratinocytes (**B**–**D**) and fibroblasts (**F**–**H**) following UVB and compound treatments. Original western blots can be found at Supplementary Figure S4. Significance was assessed by Student’s *t*-test (* *p* < 0.05, ** *p* < 0.01).

In primary keratinocytes, UVB irradiation markedly induced the phosphorylation of H2AX and PCNA transcription (Figure 2A,B), which is in agreement with the literature data [22,24]. Compounds were not able to phosphorylate H2AX (Figure 2A), suggesting that they did not act on PIKKs but directly on p53. Conversely, compound treatment induced PCNA expression (Figure 2B).

UVB treatment also significantly increased Bax expression and reduced Bcl-2 levels (Figure 2C,D). Following UVB exposure, up-regulation of PCNA and Bax in concomitance with down-regulation of Bcl-2 may enable active DNA repair in agreement with the literature [21]. However, the persistence of Bax overexpression and Bcl-2 decrease can induce cell death. Notably, compound treatment did not induce Bax (Figure 2C) or decrease Bcl2 (Figure 2D) expression.

In primary fibroblasts, UVB exposure induced H2AX phosphorylation, whereas compounds did not (Figure 2E). UVB irradiation significantly increased PCNA transcription, whereas compound treatments led to a decrease in PCNA levels (Figure 2F). A significant decrease was observed for compounds **1**–**3a**.

UVB radiation did not induce Bax expression but caused a significant reduction in Bcl-2 levels (Figure 2G,H). However, compound treatments induced Bax expression (Figure 2G) and, in general, maintained Bcl-2 at the control level (Figure 2H). Notably, compound **2b** strongly induced Bax and contemporarily reduced Bcl-2.

Thus, BTDs and TDPs exhibit different effects on DNA repair and survival pathways depending on the targeted cell type: they can be protective in keratinocytes and pro-apoptotic in fibroblasts.

### 3.3. BTDs/TDPs and Clonogenic Ability of Primary Keratinocytes and Fibroblasts

DNA replication and most DNA repair activities occur during the S-phase of the cell cycle. High levels of p21^waf1^ can be compatible with DNA repair but not with replication [25]. The incorporation of thes DNA precursor 5-ethynyl-2’-deoxyuridine (EdU), an alkyne-conjugated thymidine analog, indicates the cells that synthesize de novo DNA in the S-phase. After treatment with ionizing or UV radiation, only a fraction of cells retain their proliferative capacity. Colony-forming efficiency (CFE) assay is a cell survival test based on the clonogenic ability of single cells and assesses the propensity of each cell to maintain the proliferative potential. For keratinocyte cultures, the percentage of paraclones (i.e., keratinocytes that have undergone terminal differentiation) represents a senescence parameter [17,26].

**Figure 3:**
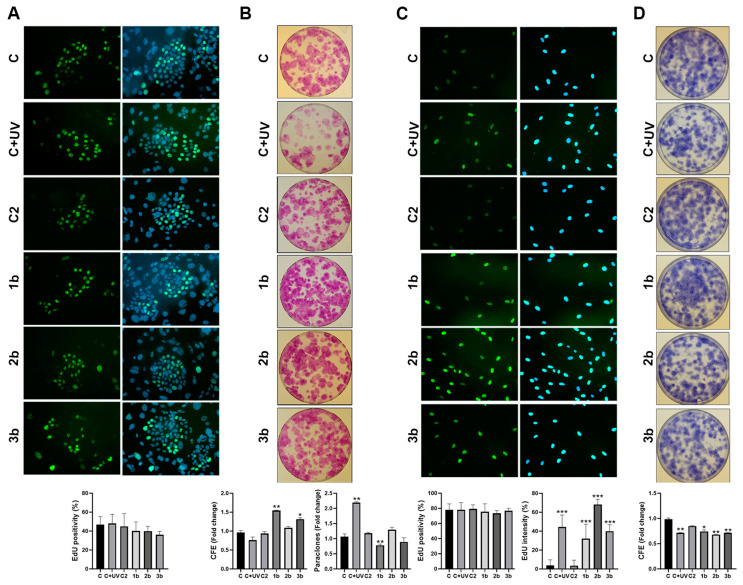
BTDs/TDPs and clonogenic ability of primary keratinocytes and fibroblasts. Representative images and values of the percentage of EdU-positive keratinocytes (20×) (**A**). Representative images of colony-forming efficiency (CFE) of keratinocytes, values of CFE, and percentage of paraclones (**B**). Representative images, values of the percentage of EdU-positive fibroblasts, and values of the percentage of more intense EdU-positive cells (40×) (**C**). Representative images of colony-forming efficiency (CFE) of fibroblasts and values of CFE (**D**). Significance was assessed by Student’s *t*-test (* *p* < 0.05, ** *p* < 0.01, and *** *p* < 0.001).

Thereafter, we treated cells only with compounds **1**–**3b**, which were the most interesting based on previous experiments. It was reported that following UVB irradiation (100 mJ/cm^2^), the distribution of keratinocytes was substantially unaltered during cell-cycle phases, whereas fibroblasts were mainly found in the S-phase [27]. In keeping with these data, no significant differences were observed in the percentage of EdU-positive keratinocytes following treatments, although the administration of **1**–**3b** slightly reduced this percentage (Figure 3A). The clonogenic ability of each treated culture was consistent with Bax and Bcl-2 expression. As expected, UVB irradiation decreased the percentage of keratinocyte CFE and increased the percentage of paraclones (Figure 3B). Conversely, treatments with compounds **1b** and **3b** induced an increase in CFE and a reduction in paraclones (Figure 3B).

No significant differences in the percentage of EdU-positive fibroblasts were observed across treatments (Figure 3C). However, increased intensity of EdU incorporation was noted in UVB- and compound-treated fibroblasts (Figure 3C), suggesting that these cells could be long-term arrested and synchronized in the S-phase for DNA repair. Notably, compound **2b** treatment exhibits a higher percentage of more intensely stained nuclei, which is consistent with its effect on Bax and Bcl2 expression. In keeping with EdU assay results, UVB exposure and compound treatments reduced fibroblast CFE (Figure 3D).

Thus, our data indicate that BTD- and TDP-treated keratinocytes retain their clonogenic ability differently from fibroblasts, strengthening findings concerning survival pathways.

### 3.4. BTDs/TDPs and Inflammation in Primary Keratinocytes and Fibroblasts

UVB radiation promotes inflammation in keratinocytes by activation of inflammasomes and successive secretion of cytokines, including IL-6, IL-1 beta, and TNF-alpha. UV exposure also stimulates the degradation of dermal ECM components [28]. In response to cellular stress, p21^waf1^ inhibits genes required for cell-cycle progression and activates several genes involved in different biological functions, such as immunosurveillance [29].

**Figure 4:**
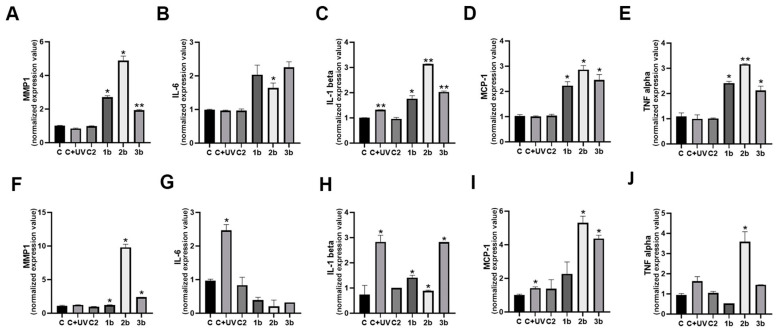
BTDs/TDPs and inflammation in primary keratinocytes and fibroblasts. Expression values of metalloprotease MMP1, IL6, IL1-beta, MCP1, and TNF-alpha transcripts in primary human keratinocytes (**A**–**E**) and fibroblasts (**F**–**J**) following UVB and compound treatments. Significance was assessed by Student’s *t*-test (* *p* < 0.05, ** *p* < 0.01).

The transcript expression of the matrix metalloproteinase MMP1 (i.e., a key enzyme for matrix remodeling) and inflammatory cytokines (i.e., IL-6, IL1-beta, MCP1, and TNF-alpha) was assessed using RT-qPCR assay (Figure 4).

In our experiments, the UVB radiation stimulated IL-1 beta expression but did not induce MMP1 or expression of other cytokines in primary keratinocytes. In contrast, BTDs and TDPs induced the expression of mediators of matrix remodeling (Figure 4A) and inflammation (Figure 4B–E). Compounds **1**–**3b** induced a significant increase in MMP1 and also primary fibroblasts (Figure 4F). Although UVB was able to stimulate cytokine expression in fibroblasts, the effect of BTDs and TDPs on inflammation was less evident compared to keratinocytes (Figure 4G–J). Notably, compound **2b** promoted MMP1 and cytokine transcription in both cell types.

Thus, BTDs and TDPs can induce MMP1 in both cell types. However, cytokines were more induced by compounds in primary keratinocytes compared to fibroblasts.

### 3.5. Effects of BTDs in a 3D Model

**Figure 5:**
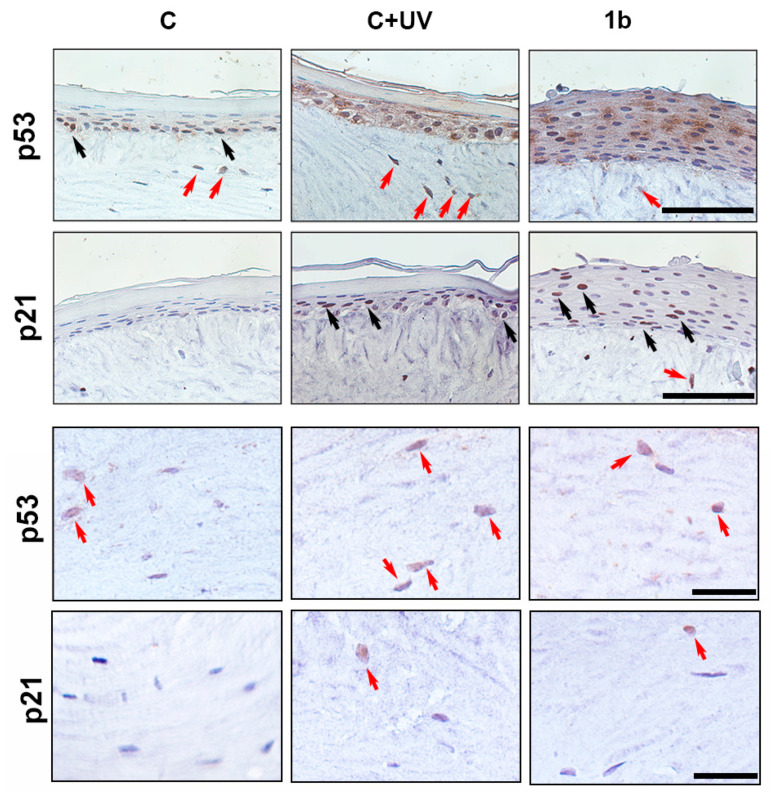
Effects of BTDs in a 3D model. Immunohistochemistry with anti-p53 and p21^waf1^ antibodies on histological preparations of skin equivalents treated with compound **1b** or exposed to UVB irradiation. Black arrows indicate the epidermal staining and red arrows the dermal staining (scale bar = 100 μm).

We investigated the activity of compound **1b**, as a selected case, by using skin equivalent 3D models, containing both a dermal equivalent and a fully differentiated epidermis. To generate the 3D models, primary fibroblasts were added to a collagen gel to recreate the dermal-like component. Primary keratinocytes were plated onto this substrate and sub-cultured for 7 days to allow the formation of the basal layers of the epidermis. Subsequently, models were sub-cultured for 7 days at the air–liquid surface to allow the generation of the stratum corneum of the epidermis. Skin equivalents were treated with compound **1b**. Skin equivalents exposed to UVB rays were used as positive controls. Skin equivalents were appropriately processed for subsequent histological analysis. The expression of p53 and p21^waf1^ was assessed by immunohistochemical assays (Figure 5).

As expected, p53 was expressed mainly in the nucleus of a few basal keratinocytes in control skin equivalents (black arrows), whereas p21^waf1^ was undetectable (C). UVB treatment induced a thickening of the stratum corneum [30] and an increase in the expression of p53 and p21^waf1^ (C + UV). Notably, p53 was expressed in the nucleus, where it acts as a transcription factor transactivating several genes, as well as in the cytoplasm, where it may trigger apoptosis and inhibit autophagy [31]. p21^waf1^ was expressed only in the nuclear compartment (black arrows), acting as a growth inhibitor [29]. The treatment with compound **1b** induced epidermal hypertrophy and an increase in p53 and p21^waf1^ expression.

Some fibroblasts in the dermal compartment expressed p53 (red arrows) of control skin equivalents. Similar to keratinocytes, p21^waf1^ expression was not observed in fibroblasts. Following UVB exposure, p53 was expressed in most fibroblasts near the epidermal compartment and p21^waf1^ in a few cells (red arrows). The treatment with compound **1b** induced both proteins in a few cells (red arrows).

Thus, compound **1b** was able to induce the p53/p21^waf1^ pathway in a 3D skin model similar to UVB exposure. These treatments stimulate keratinocyte proliferation and stratification differently from UVB.

## 4. Discussion

Most deleterious effects of solar radiation, including erythema, immunosuppression, and skin cancer, are due to UVB radiation. Although skin cancers originate from epidermal keratinocytes, the dermal microenvironment plays a key role in tumor development and progression [32].

Several DNA damaging agents (e.g., UV radiation, environmental carcinogens, anti-cancer drugs) induce the formation of bulky lesions on DNA that can be excised by the nucleotide excision repair machinery to prevent mutagenesis. UV photo-products are removed from the genome in the form of small, excised, damage-containing DNA oligonucleotides [3]. However, the fate of the removed photo-products has not been extensively examined. A growing body of evidence indicates that DNA repair side products may activate intracellular signaling pathways (e.g., MAPK and checkpoint signaling pathways), impacting the cellular response to UV and inducing immune and inflammatory responses [3,7,33].

In the present study, we examined the biological effect of novel BTDs and corresponding TDPs [16] in primary keratinocytes and fibroblasts. The treatments with a panel of chemically synthesized adducts allow for a precise and detailed examination of specific biological effects of each side-product on pathways involved in DNA damage response and tissue clearance.

We found that BTDs and TDPs activate the safeguard p53/p21^waf1^ pathway in both cell types, similar to UVB exposure. This finding suggests that excised oligonucleotides can facilitate the DNA damage response by stimulating p21^waf1^-mediated cell-cycle arrest through a feed-forward mechanism. Indeed, p21^waf1^ provides a first line of defense against dysfunctional cells that can become cancerous [34]. In addition to its role in cell-cycle arrest, p21^waf1^ is crucial for the transcription of key genes involved in the inflammatory response through transcription factors, such as SMAD and STAT [29]. By extending cell-cycle duration (“assisted cell-cycle”), p21^waf1^ provides extra time for cellular repair and/or recruitment of macrophages for immunosurveillance [35]. Stressed cells can recuperate, normalize p21^waf1^ expression, and re-enter the cell cycle. Irreparably damaged cells undergo sustained and robust p53 response, leading to apoptosis or senescence, and subsequent cytokine-mediated tissue clearance [36].

Previous studies found that the small damaged-excised oligomers bound RPA, a heterotrimeric protein complex, which binds single-strand DNA (ssDNA) with high affinity and is essential for DNA replication, recombination, and repair. The binding of RPA to p53 is reduced following UV radiation, indicating that RPA coordinates DNA repair by sensing DNA damage and releasing p53 to activate its downstream targets [37,38]. Moreover, thymine dimers activate p53, leading to nuclear accumulation, and increase the specific binding to its DNA consensus sequence as well as the transcription of p21^waf1^, PCNA, and GADD45 [12]. Similarly, BTDs and TDPs might act on the p53/p21^waf1^ axis by binding to RPA or directly to p53. Indeed, they did not induce phosphorylation of H2AX, suggesting that they can act downstream to PIKKs.

UVB response varies significantly with skin cell type [35,39], reflecting specialized functions within the tissue. Keratinocytes form the outermost layer of the skin and act as the primary barrier against UV radiation. Thus, they are more resistant to the lethal effects of UVB and remove CDPs more efficiently than fibroblasts, which are not directly exposed to solar rays but hosted in the dermal microenvironment. Notably, keratinocytes exhibit a more efficient global genome repair that acts as a backup system to remove transcription-blocking lesions [39]. Similarly, BTDs and TDPs display different effects on the survival of primary keratinocytes and fibroblasts. Our data indicate that these compounds appear to have a protective role in keratinocytes by preserving cell clonogenic ability without modulating pro- or anti-apoptotic pathways. Specifically, these compounds up-regulate the expression of PCNA, supporting DNA repair activities before re-entering the cell cycle. In addition, in the 3D model, the treatment with compound **1b** up-regulates the p53/p21^waf1^ pathway, enabling keratinocytes to maintain their clonogenic capacity, as evidenced by signs of hyperplasia. Furthermore, the compounds induce the expression of mediators of inflammation and matrix remodeling in primary keratinocytes. The reversibility of p21^waf1^-mediated cell cycle arrest and the production of cytokines by compounds can have a key role in increasing clonogenicity and reducing paraclones. However, this induction of inflammatory mediators in keratinocytes might have a long-term negative effect in vivo. Small excised and damaged DNA products of NER were found following UV exposure of ex vivo skin models at doses that led to minimal erythema and were stable for at least 12 h [7]. Therefore, after excessive sun exposure, slow or inefficient degradation of damaged excised oligonucleotides might contribute to the early stages of skin tumorigenesis or photosensitive skin disorders by impacting inflammatory and immune signaling pathways. Of note, photosensitivity is often an early manifestation in systemic lupus erythematosus patients, characterized by mutations in enzymes responsible for ssDNA degradation [3].

It has been reported that excised CPD-containing DNA is released from UVB-irradiated keratinocytes through extracellular microvesicles in the microenvironment and can be taken up by neighboring cells through a bystander effect. These findings provide a mechanism by which UVB-damaged oligonucleotides can be transmitted from photoexposed to non-photoexposed cells, such as fibroblasts, with possible systemic effects of UVB exposure throughout the body [33]. In contrast to keratinocytes, BTDs and TDPs down-regulate PCNA and induce pro-apoptotic effects on fibroblasts, which do not retain their clonogenic ability. Moreover, their effect on inflammation is less evident. Notably, these compounds significantly increase the expression of IL-1 beta and MMP1, which can remodel the dermal microenvironment. Such activity from fibroblasts could promote proliferation and invasion of mutated keratinocytes, potentially favoring tumor development.

UV response also depends on the type of damage [40]. An example of the specific and different biological effects of each compound is evident in the cellular response to compounds **1b** and **2b**. Although both compounds activate the p53/p21^waf1^ safeguard pathway, they exhibit different effects in both cell types. In keratinocytes, compound **1b** displays a pronounced pro-survival effect leading to a substantial increase in clonogenic capacity and cytokine expression, alongside a marked reduction in paraclones. On the other hand, compound **2b** also provides a protective role in keratinocytes but preserves clonogenicity at levels comparable to the control and does not significantly impact the percentage of paraclones. However, compound **2b** induces a strong inflammatory response and MMP1 expression. Conversely, in fibroblasts, compound **1b** demonstrates a slight pro-apoptotic effect as indicated by the moderate increase in Bax and Bcl-2 expression and the decrease in clonogenic ability. Moreover, it significantly elevates only IL-1 beta and MMP1 levels. On the other hand, compound **2b** exhibits a strong pro-apoptotic action by significant up-regulation of Bax and down-regulation of Bcl-2, leading to a substantial decrease in clonogenic ability. Additionally, compound **2b** significantly increases the levels of key cytokines involved in inflammatory and immune responses, such as MCP1, TNF alpha, and IL1 beta, as well as MMP1.

## 5. Conclusions

The results of the present study are consistent with previous data on simple TDs [12,14,15] and shed light on the differential action of several new compounds.

Overall, our findings indicate that novel BTDs and TDPs can act in the short term as safeguard mechanisms, helping DNA damage response in both primary keratinocytes and fibroblasts. Furthermore, they have distinct biological effects that depend on damage form and cell type.

Whether the persistence of BTDs and TDPs, which mimics chronic sun over-exposure or defective oligomer processing, might play a role in photosensitive disorders and skin tumorigenesis, remains a challenge for future studies.

## Data Availability

The original contributions presented in this study are included in the article/Supplementary Materials. Further inquiries can be directed to the corresponding authors.

## Supplementary Materials

The following supporting information can be downloaded at https://www.mdpi.com/article/10.3390/biom14121484/s1: Figure S1: Toxicity of BTDs and TDPs in primary keratinocytes. Toxicity was evaluated by MTT assay after 24 h and 48 h of treatment with compounds **1a**–**3a** and **1b**–**3b** in primary human keratinocytes using 50 and 100 μM concentrations. Figures S2–S4: Original Images for Blots. Table S1: Primer sequences for the genes were assessed using RT-qPCR.
