# Supplementary material for: Differential Effects of Biomimetic Thymine Dimers and Corresponding Photo-Adducts in Primary Human Keratinocytes and Fibroblasts
Source: Biomolecules. 2024 Nov 21;14(12):1484. doi: 10.3390/biom14121484 (PMC11726716; doi:10.3390/biom14121484)
Supplement: Supplementary file 1 [file biomolecules-14-01484-s001.zip › biomolecules-3248187-supplementary.pdf]

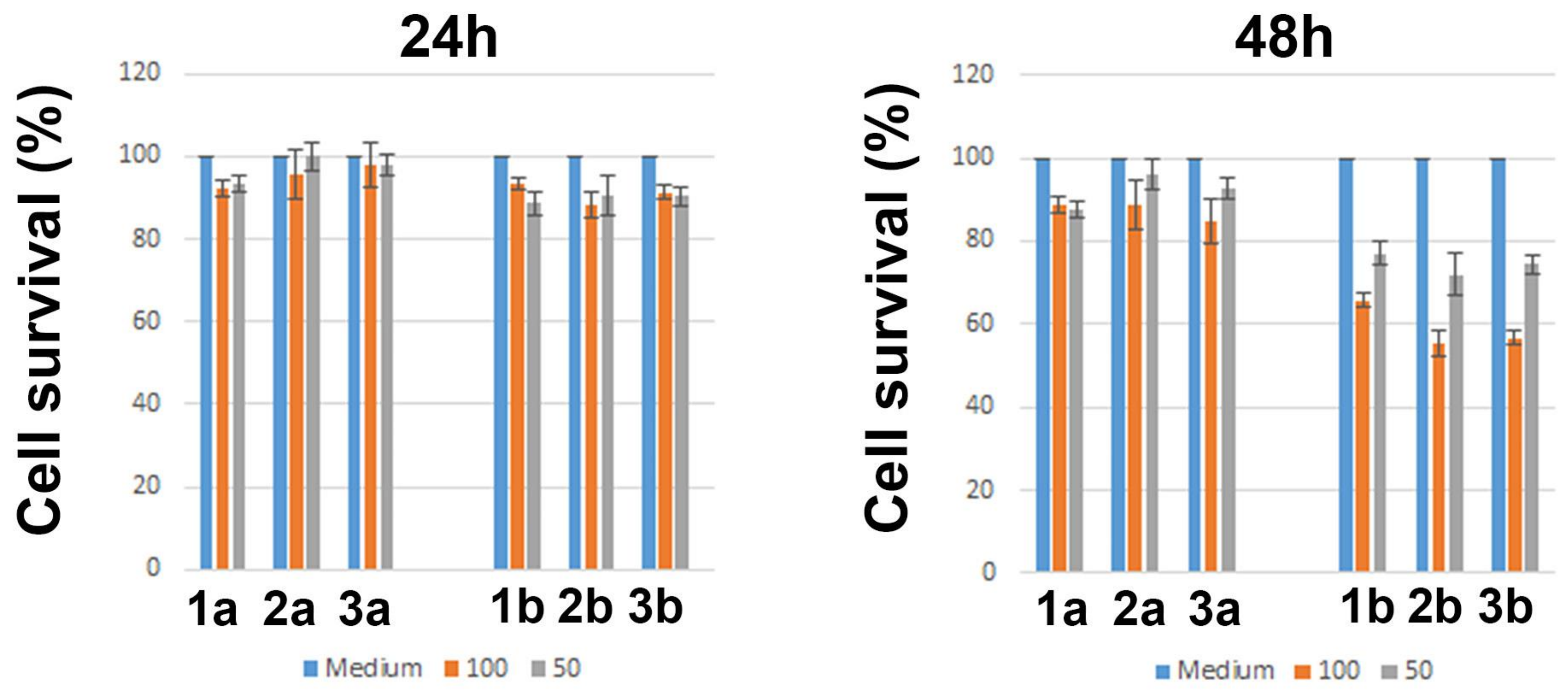

Figure S1. Toxicity of BTDs and TDPs in primary keratinocytes. Toxicity was evaluated by MTT assay after 24h and 48h of treatment with compounds 1a-3a and 1b-3b in primary human keratinocytes using 50 and 100uM concentration.

Figure S2

Uncropped blots of Figure 1D

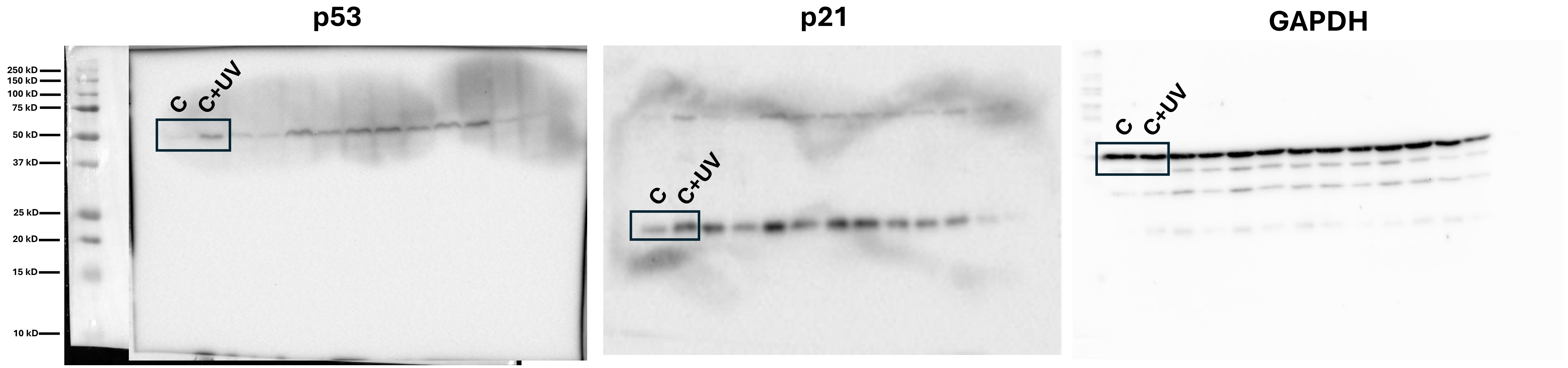

Uncropped blots of Figure 1E

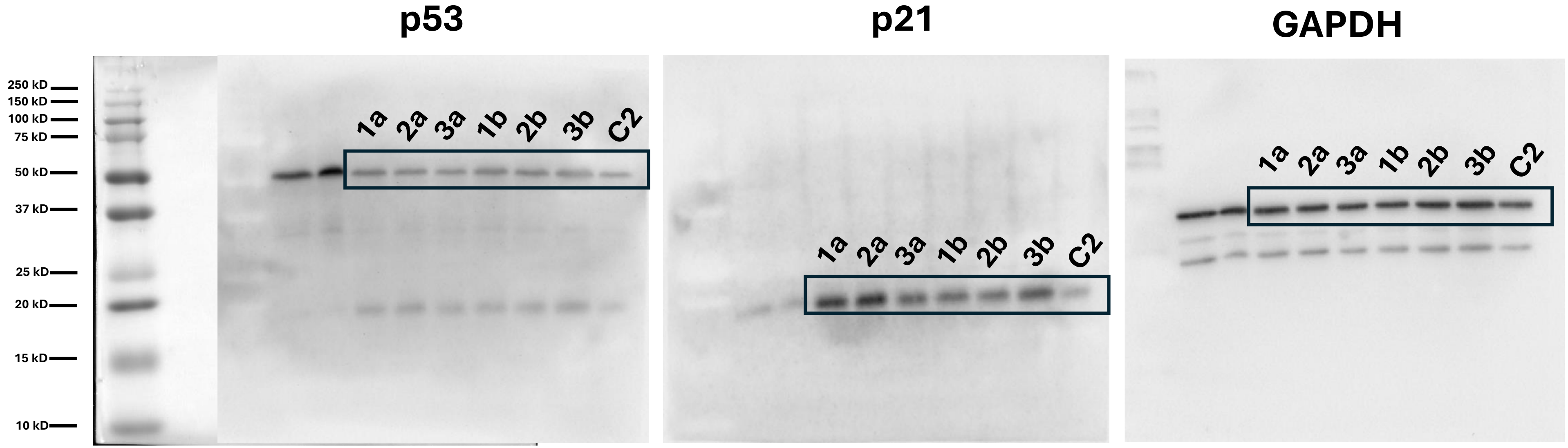

Figure S3

Uncropped blots of Figure 1H

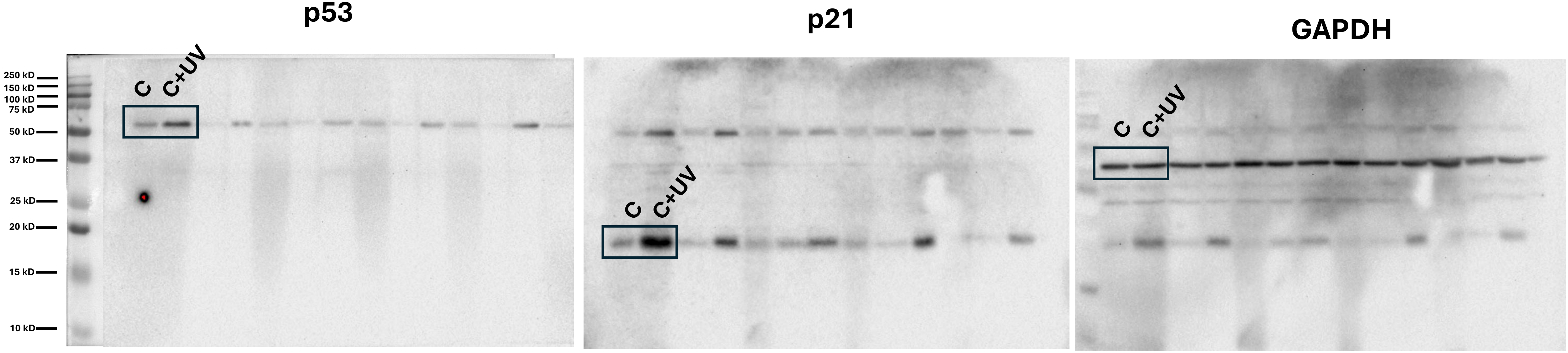

Uncropped blots of Figure 1I

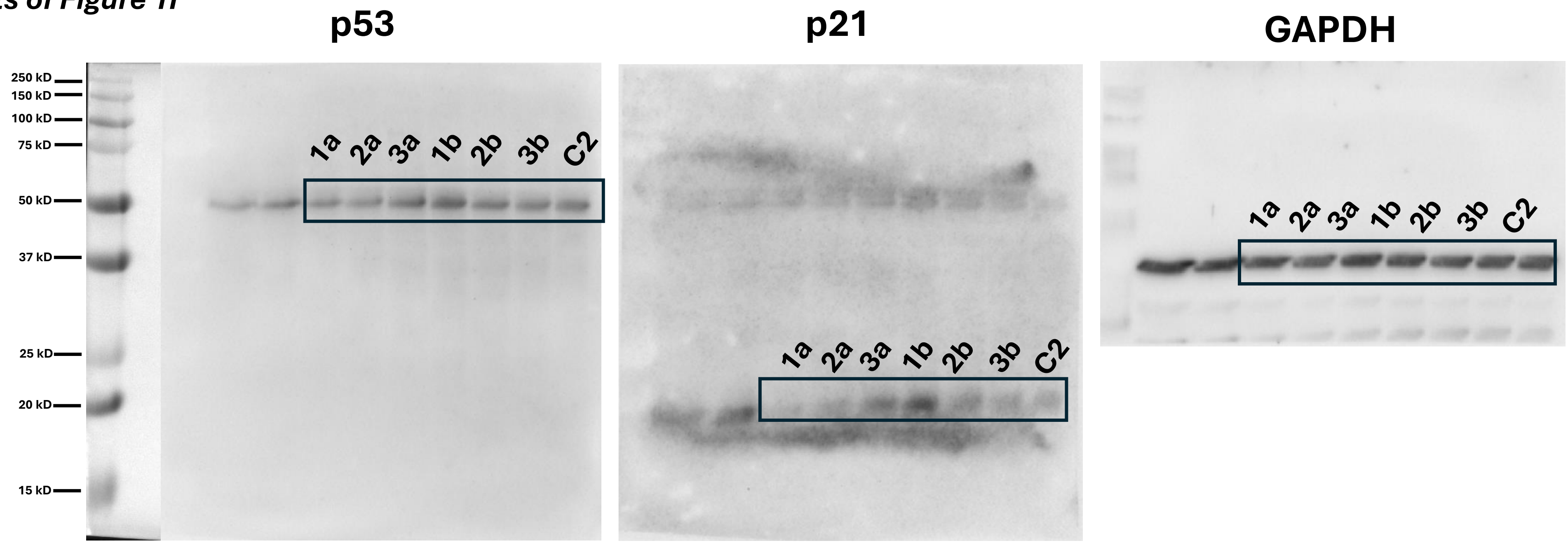

Figure S4

Uncropped blots of Figure 2A

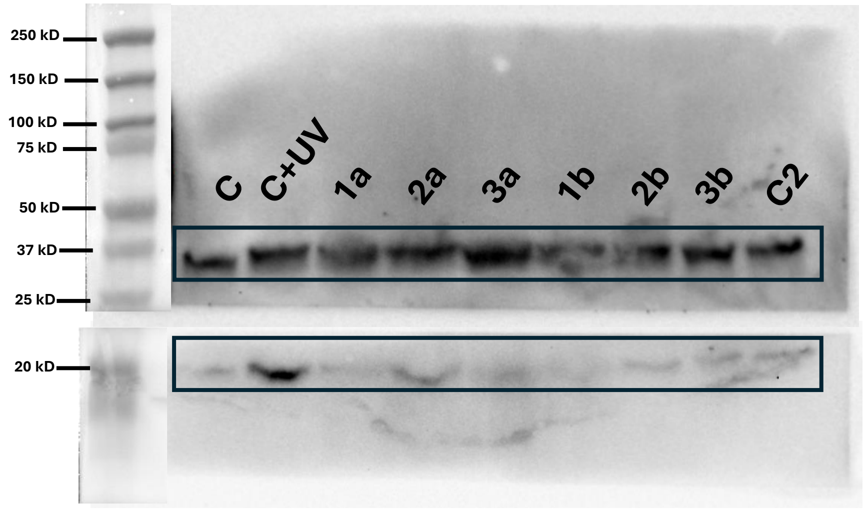

Uncropped blots of Figure 2E

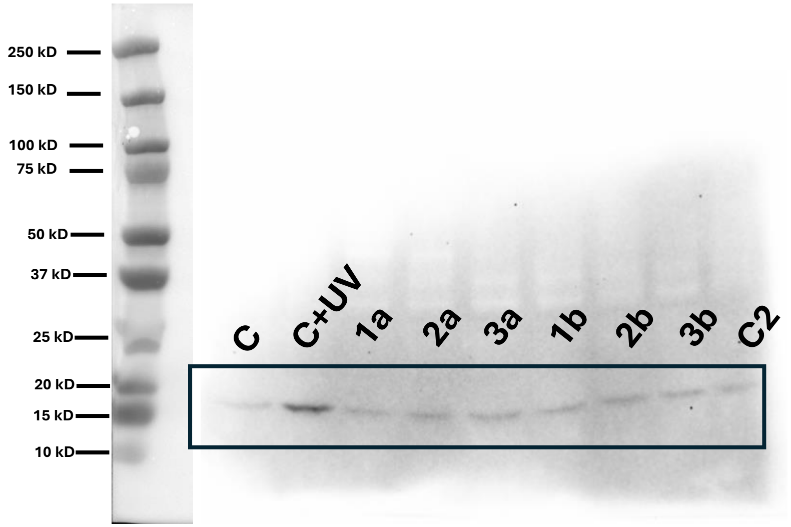

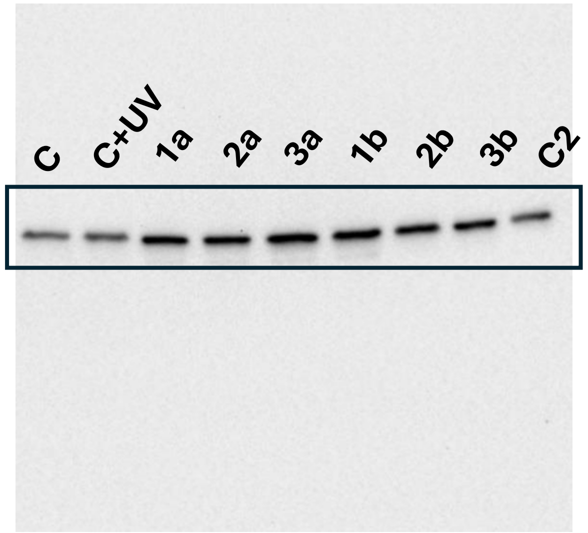

Table S1. Primer sequences for the genes assessed using RT-qPCR

mRNA	*Forward*	*Reverse*
P53	*5' TCAACAAGATGTTTTGCCAACTG 3'*	*5' ATGTGCTGTGACTGCTTGTAGATG 3'*
P21^{waf1}	*5' TGTCTTGTACCCTTGTGCCTC 3'*	*5' GAGAAGATCAGCCGGCGTTT 3'*
PCNA	*5' CAAGTAATGTCGATAAAGAGGAGG 3'*	*5' GTGTCACCGTTGAAGAGAGTGG 3'*
Bax	*5' CGCCCTTTTCTACTTTGCCA 3'*	*5' GTTCTGATCAGTTCCGGCAC 3'*
Bcl2	*5' GCCCTGTGGATGACTGAGTA 3'*	*5' GAAATCAAACAGAGGCCGCA 3'*
MMP1	*5' TGTGGTGTCTCACAGCTTCC 3'*	*5' CGCTTTTCAACTTGCCTCCC 3'*
IL6	*5' CAATCTGGATTCAATGAGGAGAC 3'*	*5' CTCTGGCTTGTTCCTCACTACTC 3'*
IL1 beta	*5' GCCCTAAACAGATGAAGTGCT 3'*	*5' ACCAGCATCTTCCTCAGCTT 3'*
MCP1	*5' GATCTCAGTGCAGAGGCTCG 3'*	*5' TTTGCTTGTCCAGGTGGTCC 3'*
TNF alpha	*5' TCTTCTCGAACCCCGAGTGA 3'*	*5' TCTTCTCGAACCCCGAGTGA 3'*
GUSB	*5' TGCAGGTGATGGAAGAAGTG 3'*	*5' TTGCTCACAAAGGTCACAGG 3'*
